# Septal reduction therapy in obstructive hypertrophic cardiomyopathy: contextualizing outcomes in an era of cardiac myosin inhibition

**DOI:** 10.1093/ehjopen/oeag087

**Published:** 2026-05-22

**Authors:** Anirudh Govind

**Affiliations:** Jawaharlal Institute of Postgraduate Medical Education and Research (JIPMER), Dhanvantri Nagar, Pondicherry 605006, India

**This correspondence refers to “Long-term outcomes after septal reduction therapy for obstructive hypertrophic cardiomyopathy: a nationwide cohort study” by A. Schelldorfer *et al.*, https://doi.org/10.1093/ehjopen/oeag053.**

**The author’s response is available at: https://doi.org/10.1093/ehjopen/oeag088.**

The nationwide cohort study by Schelldorfer *et al*.^1^ provides population-level data on long-term outcomes following septal reduction therapy (SRT) for obstructive hypertrophic cardiomyopathy (HCM) in Switzerland. The study's two principal findings merit careful examination: that SRT was associated with a substantially lower incidence of the composite primary endpoint compared with hospitalized patients with non-obstructive HCM (HR 0.17; 95% CI 0.07–0.42), and that outcomes following SRT were comparable to those of age- and sex-matched surgical controls without cardiac disease (HR 1.53; 95% CI 0.56–4.18). These are clinically important observations, particularly given the evolving landscape introduced by cardiac myosin inhibitors. I wish to offer four reflections that may further contextualize and extend the authors’ conclusions.

First, the non-obstructive HCM control group likely represented a more advanced population, with ∼30% carrying a concurrent heart failure diagnosis. This compositional asymmetry complicates interpretation of the HR of 0.17, a magnitude exceeding that observed in cohorts with less selection for advanced disease.^2^ The effect estimate likely reflects ascertainment bias: identifying non-obstructive HCM exclusively through inpatient hospitalizations selects for sicker patients. Moreover, obstructive and non-obstructive HCM are pathophysiologically distinct entities with divergent natural histories, and heterogeneity in baseline risk persists even after matching.

Second, stratification by SRT modality was precluded by sample size. While long-term survival is broadly comparable between surgical myectomy and transcoronary ablation of septal hypertrophy (TASH), propensity-matched data consistently demonstrate higher pacemaker implantation and reintervention rates following TASH,^3^ and pooling both modalities may obscure clinically meaningful heterogeneity. The roughly equal procedural distribution in this cohort makes this gap especially pertinent.

Third, excluding patients who died during the index hospitalization introduces survivor bias, underestimating early procedural risk, particularly for myectomy at non-specialist centres. The well-documented volume-outcome relationship for myectomy, with substantially higher in-hospital mortality at low-volume centres,^4^ renders population-level administrative data particularly vulnerable when SRT is dispersed across centres of varying expertise, as in Switzerland.

Fourth, this study predates the widespread availability of mavacamten and aficamten. The VALOR-HCM trial demonstrated that cardiac myosin inhibition eliminated the SRT indication in a substantial proportion of referred patients.^5^ The finding that post-SRT outcomes approach those of a healthy surgical population thus provides a valuable benchmark against which pharmacological gradient reduction must be measured. Crucially, myosin inhibitors do not modify the underlying sarcomeric pathology; whether sustained therapy reduces composite endpoints over decadal time horizons remains to be established.

Notwithstanding these limitations, this study makes a valuable contribution by providing the first population-based Swiss data on post-SRT outcomes, establishing a national benchmark at a critical inflection point in obstructive HCM management.

## Data Availability

No new data was generated in this manuscript. It is only a response to a previously published article.
